# An LBS and agent-based simulator for Covid-19 research

**DOI:** 10.1038/s41598-022-25175-5

**Published:** 2022-12-08

**Authors:** Hang Du, Zhenming Yuan, Yingfei Wu, Kai Yu, Xiaoyan Sun

**Affiliations:** School of Information Science and Technology, Hangzhou Normal University, Hangzhou, 310016 China

**Keywords:** Epidemiology, Psychology and behaviour, Engineering

## Abstract

The mobility data of citizens provide important information on the epidemic spread including Covid-19. However, the privacy versus security dilemma hinders the utilization of such data. This paper proposed a method to generate pseudo mobility data on a per-agent basis, utilizing the actual geographical environment data provided by LBS to generate the agent-specific mobility trajectories and export them as GPS-like data. Demographic characteristics such as behavior patterns, gender, age, vaccination, and mask-wearing status are also assigned to the agents. A web-based data generator was implemented, enabling users to make detailed settings to meet different research needs. The simulated data indicated the usability of the proposed methods.

## Introduction

The urgency of Covid-19 pandemic has attracted the attention of many researchers, such as the prediction of virus transmission^1, 2^, the impact of the epidemic on travel^3^, and the impact of Non-pharmaceutical interventions on Covid-19 transmission^4^. We notice that this research used human mobility data. However, out of concern about disclosing citizens' privacy^5^, not all researchers are authorized to collect and use human mobility data. This has caused considerable difficulties for relevant research.

Compartmental models^6–8^ and Cellular Automata (CA)^9, 10^ are the traditional models of infectious disease transmission. The use of compartmental models and CA to model populations has been reported to account for 78% of all relevant studies for Covid-19^11^. However, compartmental models usually ignore the influence of individual characteristics as well as geography, and CA lacks the potential for expressing interactions and movements among individuals. Compared with Compartmental models and CA, the Agent-based Model (ABM) can better represent individual behaviors and interaction between individuals. After utilizing geographical data such as land use and population distribution, the synthetic agents can express the heterogeneity of both population and the built environment. For instance, Zhou^12^ et al. proposed a framework using Bayesian networks and generalized raking to produce spatially detailed and heterogeneous synthetic populations in Singapore, which outperforms traditional population synthesis methods. However, much research only modeled the agents in a static way without detailed activity and trajectory chain^12, 13^, which is crucial for contact tracing and the corresponding Nonpharmaceutical interventions (NPIs) simulation. Many population travel studies are trying to estimate or extract population origin–destination matrices^14^ or daily activity chains^15–17^, which are relatively high level results. In some studies, daily activity patterns for agents were assigned according to survey samples or empirical data^18, 19^, therefore with limited diversity among agents. Moreover, many of the agents in the simulations for Covid-19 do not move according to the real road network. For example, Giacopelli^20^ et al. proposed a full-scale agent-based model in the Lombardy region of Italy, where agents move according to random walk approach in their study. In the study of Goldenbogen^21^ et al. weekly schedules were assigned to determine the agent’s presence at each hour, but no detailed trip trajectories between places were generated. In particular, public transportation, which plays significant role in virus transmission, are missing in the simulation. On the other hand, the temporal resolution of many studies is modest, often an hour^21^ or even a day^22^, which leads these studies to ignore the transmission of the virus after a short exposure. Furthermore, We noticed that most of the synthetic mobility data only recorded the locational information of the trajectories and without the demographic characteristics of individuals^23^ or with only basic demographic traits (age, gender, home location and housing)^24^. For epidemiological simulation, however, more disease transmission-related features are required. All these factors limited researchers from conducting more detailed studies.

To address these problems, this paper proposed an agent-based method for generating pseudo population mobility data with Location-based Service (LBS). LBS is a service that uses location-aware technology to sense the user's location information and provides various services^25^, such as Google Maps API provides information including maps, places and routes. Unlike traditional geographic information systems (GIS), LBS can acquire data through context-aware services^26^, which are particularly suitable for dynamic scenarios^27^. For example, Cui^15^ et al. used the Google Places API to acquire the types of commonly used locations for generating daily activity plans. Research suggested that the mobility of a population could be divided into several movement patterns^23, 28^. If these patterns was linearly combined, it was possible to simulate the main human mobility at the urban scale^16^. Our method aimed to generate human mobility data with high spatial and temporal resolution based on agent-specific activity schedules for Covid-19 simulation.

The main contributions of this paper are:

- A method for generating pseudo human mobility data on a per-agent basis was proposed for Covid-19 simulation, by combining group pattern settings and agent-specific activity chain generation.
- The information provided by LBS is used to generate agents’ mobility data conforming to the real road network and the data was processed into GPS-like format with high spatial and temporal resolutions.
- In addition to agent mobility data, information such as age, gender, vaccination and mask-wearing status was also provided for each generated trajectory for a purpose of better virus transmission simulation.
- Based on the proposed method, a web-based generator was also implemented, which can be used by researchers to generate mobility data flexibly without any code-level changes.

## Methods

**Figure 1:**
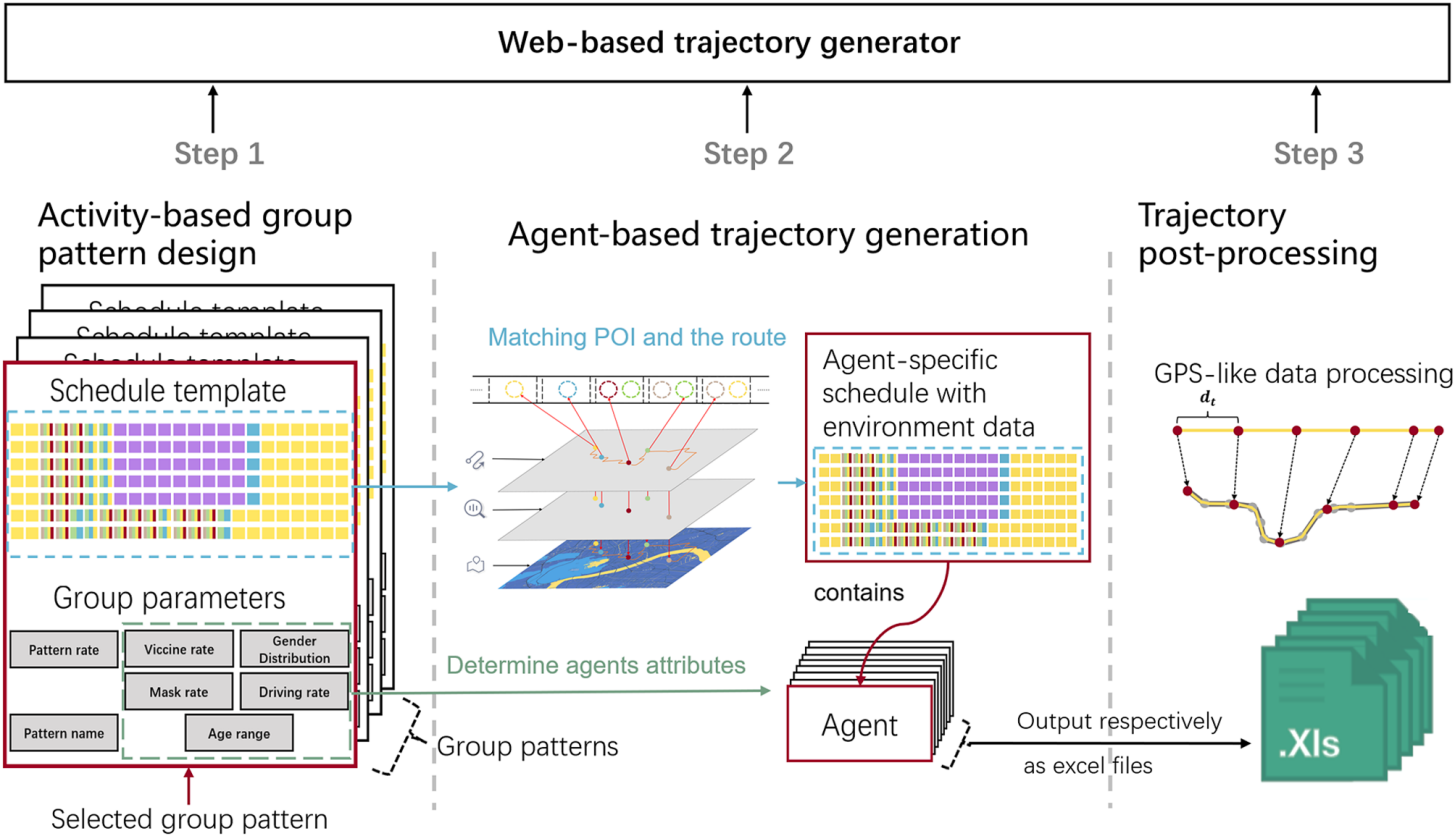
Overview of the proposed methods. Three steps are used to describe our proposed method.

Our proposed method could be roughly divided into three steps (see Fig. 1):

- Activity-based group mobility pattern design.
- LBS-based agent trajectory generation.
- Data processing for GPS trajectories.

### Activity-based group mobility pattern design

We defined the group pattern to determine the activities and attributes of each individual who belonged to the group, which contains a set of parameters as well as a schedule template. Since human mobility data consists of multiple individuals with different behavioral characteristics, it was challenging to distill individual-level statistical patterns from such aggregated data^29^. Instead, it had been well reported that we could use ABM to model human mobility data from the bottom-up by dividing agents into several groups with heterogeneous behavioral patterns^26, 28^. Therefore, we set the following parameters for the group patterns (see Table 1). Among them, group name and group probability were parameters of the group itself. Virus infection related attributes could influence the transmission and development of Covid-19, including vaccination^30^, mask^31^, age^32^, and gender distribution^32, 33^. Meanwhile, mobility related attribute, i.e., driving preference was also included as a group parameter that could influence an agent's travel choice as well as one’s infection probability during the trip.

**Table 1:** Parameters of the group pattern.

Type	Parameters	Description
General attributes	Group name	name of the group pattern
	Group probability	the percentage of an agent belongs to the group (%)
Virus infection related attributes of agents in the group	Vaccination rate	Determine the vaccination rate (%)
	Mask rate	Determine the mask-wearing rate (%)
	Age range	Determine the age range (in years)
	Gender distribution	Determine the gender distribution (integer)
Mobility related attribute of agents in the group	Driving preference	Determine agents' driving preferences (integer)

**Figure 2:**
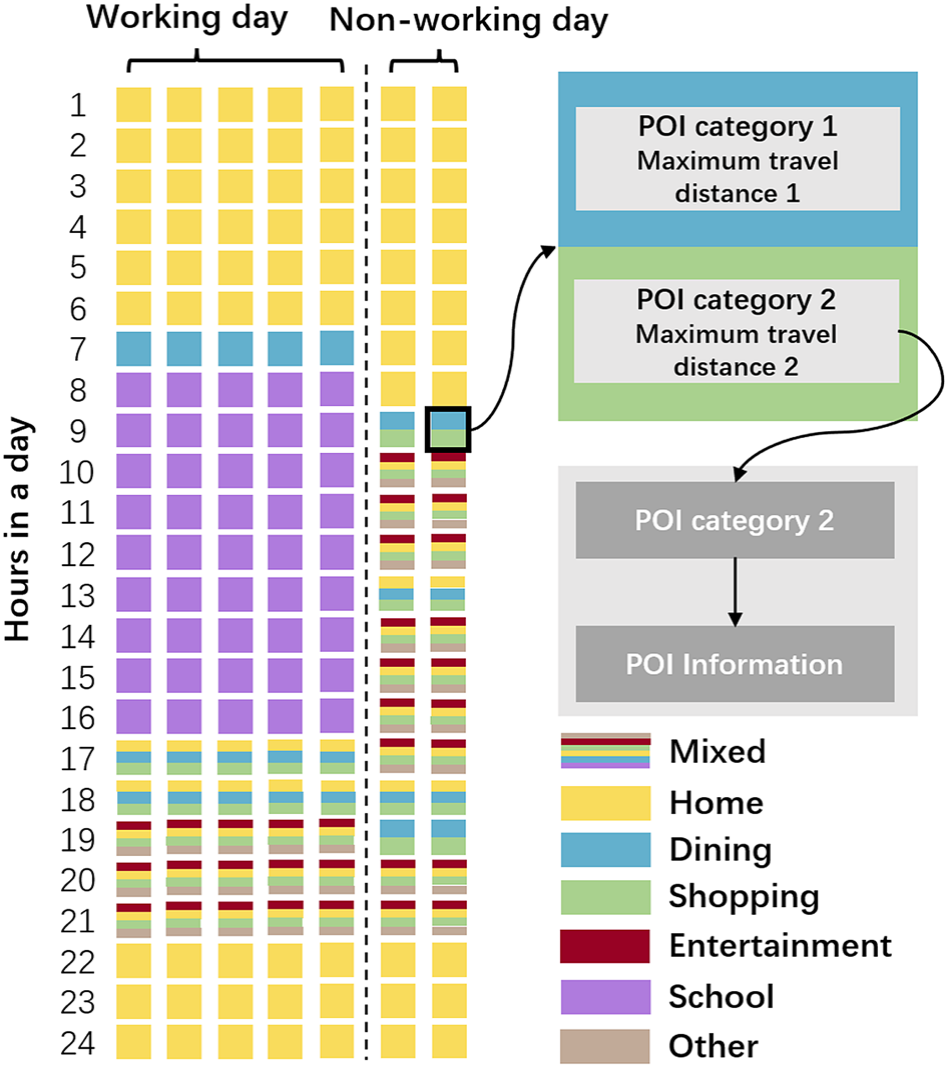
Schedule template for a certain group (student group). The different colored squares (brown square, green square, yellow square, etc.) represent an hour in the schedule template. For each square, there will be one or more categories inside, which represent the POI categories that such agent needs to go to in that hour. we use colors to distinguish the categories of places.

A schedule template (see Fig. 2) was assigned to all agents who belong to this group. The schedule template was used to constrain the activities of all agents in the group, which was assumed to be known to the user. The time duration of the schedule template was one week and could be divided into two categories: working days and non-working days. By default, Monday to Friday were working days, and Saturday and Sunday were non-working days. The time resolution for each day was one hour. The schedule template specified the activity for the agents during a certain time window, in a form of categories of target places and the maximum travel distance. Be notice that for each agent in the same group, even though they all followed the schedule template, the target places they went to could be different, which resulting in different arrival times and thus affecting their following mobility activities. Therefore, each agent had its agent-specific daily activity chains when LBS information was acquired (see detail in the following session).

Considering the fact that the virus transmission was demographically heterogeneous (such as age and gender^23^), e.g., Covid-19 was significantly more lethal for older people than other populations^34^, we also assigned epidemic correlated attributes in the group pattern. These attributes were vital for virus transmission simulation, but missing in traditional human mobility data such as mobile phone location data and GPS data. The attributes of agents are listed in Table 2, whose values were affected by the corresponding parameters in the group pattern.

**Table 2:** Parameters of the agent.

Parameters	Format	Description
Group name	String e.g. ordinary worker	The name of the group pattern to which the agent belongs
Age	Integer e.g. 37	The age of the agent
Gender	Text e.g. FM	The gender of the agent
Mask rate	Integer e.g. 5	The probability of wearing a mask per trip
Vaccination status	Integer e.g. 5	individual vaccination status
Driving preference	Integer e.g. 3	Decide on agents' driving preference

### LBS-based agent trajectory generation

**Figure 3:**
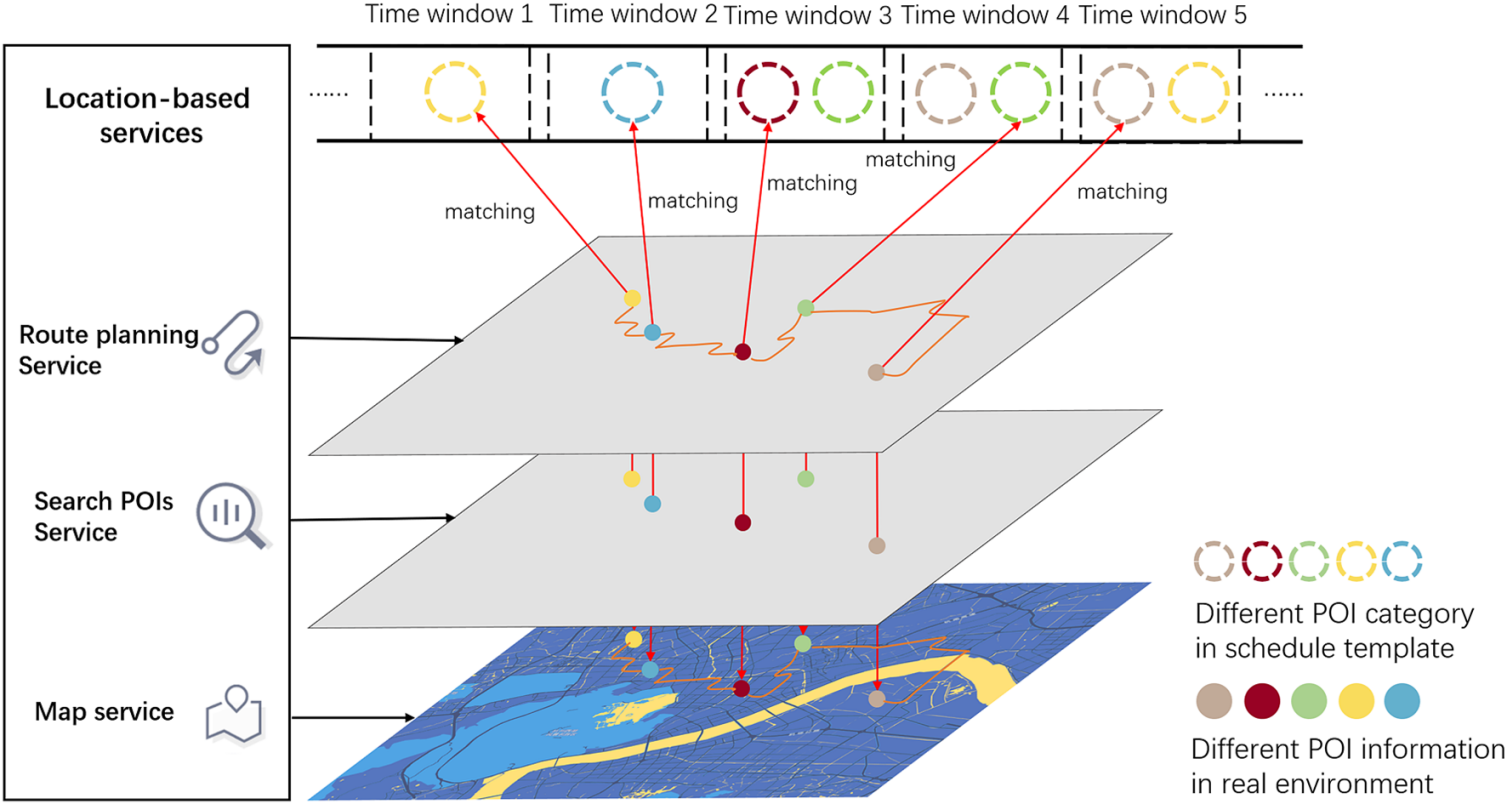
Generating agent-specific schedule with LBS. According to the order of the schedule template, send a request to the LBS provider (which provides the search POIs service), match the POI information to the schedule template, and use the obtained POI information to continue to send a request to the POI provider (which provides the route planning service) to get the corresponding trajectory data. Repeat these steps to generate an agent-specific schedule after traversing the entire schedule template, which is no more extended POI categories but factual POI information.

With the pre-set group pattern, our method generated agent-specific schedules for each agent in this step. We generated the trajectory dataset based on the schedule template on a per-agent basis in two steps (see Fig. 3): (1) determined a target place within the Place of Interest (POI) category as defined in the schedule template using POIs search service to build an agent-specific schedule. (2) generating trajectories according to the agent-specific schedule, with the route planning services. We send a request to Search POIs Service to get a list of POIs returned by the LBS provider based on the POI category and maximum distance specified in each agent's schedule template. Currently, we randomly select one of the eligible POIs and assign it to the schedule template. Repeating this step, we could generate an agent-specific schedule based on the schedule template but with different destinations. The agent-specific schedule now contains factual geographic information.

After generating the agent-specific schedules, we used the route planning service of the LBS provider to retrieve the corresponding data. By setting parameters such as origin and destination, travel mode, and estimated departure time, we retrieved the planned route that matches the road networks and accessibility. For travel mode, we considered driving as an independent mode with almost zero possibility for infection during the trip. Different driving preference rates were assigned to each group pattern. Meanwhile, the probability of driving was also affected by the travel distance. For each travel, the probability of driving was computed as following:

1 $$\begin{array}{c}p\left(d\right)=\frac{2}{1+{e}^{-\mathrm{\alpha }{p}_{v}\frac{d}{D}}}-1\end{array}$$

where the probability function $p\left(d\right)$ received the distance $d$ between the origin and destination as an input. $D$ was the distance threshold, which we currently set to 5000 m. ${p}_{v}$ was the driving preference parameter set in the group pattern. $\mathrm{\alpha }$ had a value of 0.4, making the probability of driving close to 100% when ${p}_{v}$ equals 10. Other travel modes apart from driving were considered as modes with infection probabilities, such as walking, cycling, and public transportations, whose probability were determined in a similar way. Equation (1) is also used to determine the probability of traveling by public transportation, cycling, walking, etc. (with different D).

Although agents in the same group share the same schedule template on a week basis, when different POI and transportation choice was selected, agent-specific schedules were generated on an individual basis. Especially for route planning services, routes matching the local road conditions and the operation of public transportation could also be achieved. Therefore, the diversity of the generated data could be expected, which made the biggest differences between our method and earlier works. Compared to traditional sources of population mobility data, the data provided by using LBS is pre-processed, which eliminates the need for map matching^35^. Depended on the service area of the LBS provider, data in large geographical area (province, country level) could be generated accurately. In addition, LBS data was cloud-based, therefore the requirement of local computing resources was reduced, as we only need to perform simple processing and stitching operations on the received data.

### Data processing for GPS trajectories

**Figure 4:**
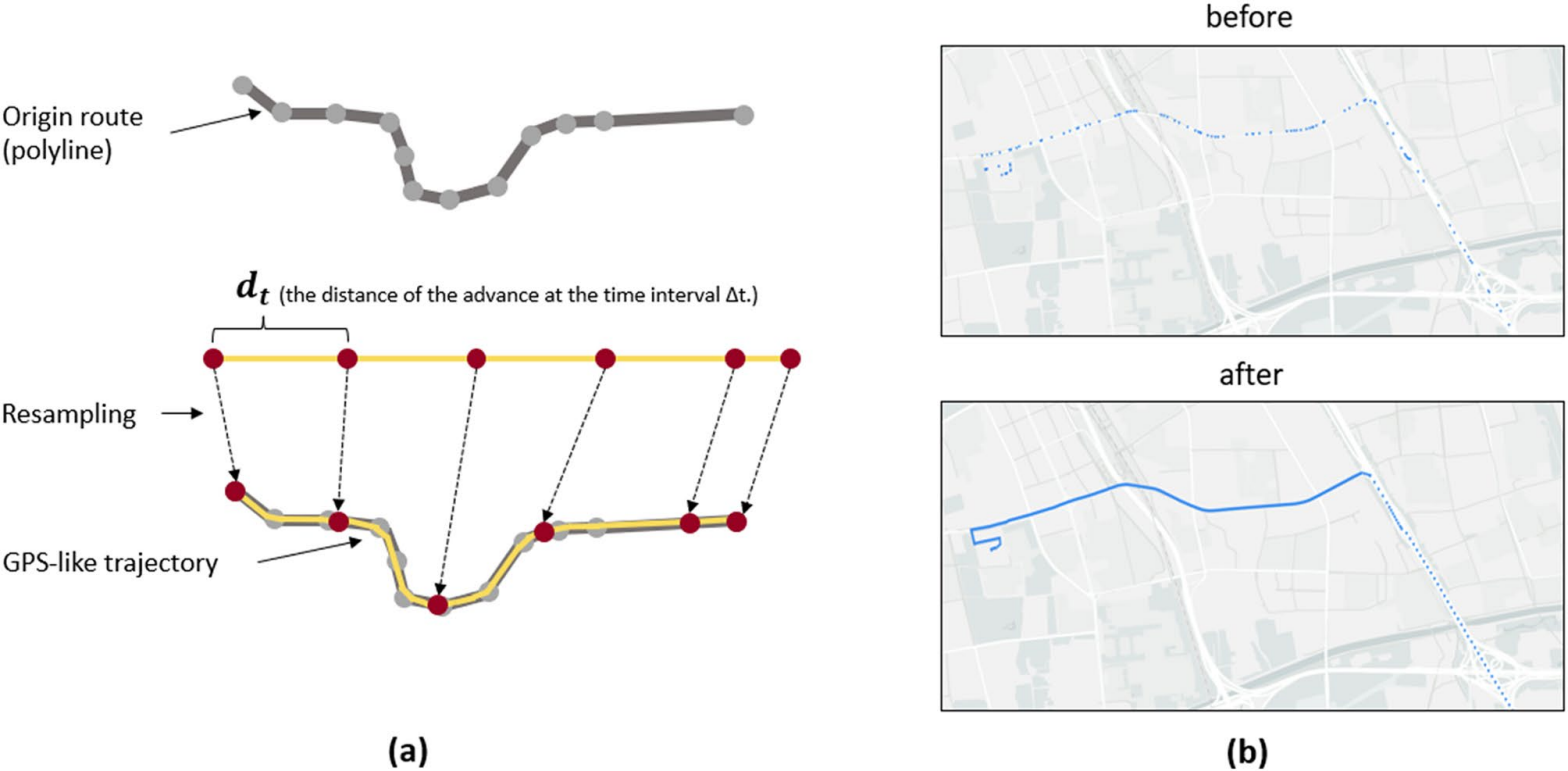
Resampling process (**a**) and the comparison before and after resampling (**b**). (**a**) shows how the original route is resampled. (**b**) before and after compare the effect after resampling (created by Gaode Map JS API, version 2.0, https://lbs.amap.com/api/jsapi-v2/summary). The blue dots represent the data points in the route.

After the first two steps, we generated the trajectory data for each trip according to agent-specific schedules, however, these trajectories returned from the LBS provider were low-resolution and only as a route indicator when no turning required between any two adjacent points on the route (see Fig. 4b). In addition to traditional travel survey^36^, human mobility data currently used for Covid-19 research were multi-sourced and multi-faceted^29^, including trip records from the Automated Fare Collection (AFC)^28^, Call Detail Records (CDRs) from mobile network operators^14, 37^, location data from the Global Positioning System (GPS)^15^ and geotagged data from social media^38^, etc. Among them, travel survey was time-consuming and often require further extraction^37^. Geotagged data from social media were discontinuous and had the problem of incomplete population coverage^38^. GPS data were accurate enough and could be collected with mobile devices continuously in a daily basis, therefore, post-processing was performed to generate GPS trajectory data for each trip segments (POI to POI).

For each segment, we obtain the polyline of the trajectory and the distance $\overrightarrow{d}$ of the segment, and the time spent $t$ from the LBS. We could then calculate the average velocity $\overrightarrow{v}$ of the segment. After we set the time interval $\Delta t$ for sampling, we could obtain the distance $\overrightarrow{{d}_{t}}$ of the advance at the time interval $\Delta t$.

2 $$\begin{array}{l}\overrightarrow{v}=\frac{\overrightarrow{d}}{t}, \end{array}$$

3 $$\begin{array}{l}\overrightarrow{{d}_{t}}=\overrightarrow{v}\times \Delta t\end{array}$$

With the $\overrightarrow{{d}_{t}}$ calculated, we could then compute the distance and resample each point in the original route in sequence (See Fig. 4a for a schematic of the process). Studies reported that GPS data with a data interval of 5 s reduces data storage requirements without compromising usability and accuracy^39^. Therefore, we set ∆t to 5 s. We could find that the resampled points were denser after the resampling process and could accurately reflect the segments' speed change (See Fig. 4b).

### Web-based data generator

To facilitate the setting of the parameters of the group pattern as well as the schedule template, we implemented a web-based data generator with a Vue.js frontend and a Spring Boot backend. We chose Gaode Open Platform as our LBS provider because of its good usability in China. A convenient interface for setting was provided, and the usage details were shown in Supplementary Fig. S1a–c. We generated the dataset with the generator and export each agent's data as an excel file, including the agent's attributes and all the trajectories generated according to the assigned time range. Various types of information for each time spot were recorded, as shown in Supplementary Table S1. Our method could also provide information about places and individuals in addition to the GPS trajectory data, which was crucial for research on the spread of infectious diseases.

## Results

We set six different group patterns using the data generator to validate our method, including general worker, overtime worker, freelancer, middle school student, pupil, and retiree. The detailed parameter settings was shown in Supplementary Table S2. Ordinary worker had the most enormous group rate of 40%, followed by retiree at 25%. The parameters such as group rate and vaccination rate were set according to the census data of the city. Except for retiree and freelancer, the schedule templates for the rest of the groups distinguish between working days and non-working days. We set these groups to travel more intensively on working days, including pupils and middle school students. One of the reasons we did not consider setting the college student group is that not all cities have college students, and college students tend to be confined to the interior of the campus and have more specific behavior patterns^40^.

Nonpharmaceutical interventions (NPIs) had been employed worldwide to stop the rapid spread of Covid-19. NPIs were grouped into 3 major categories: school closure; cancellation of public gatherings; and isolation and quarantine^41^. We set four levels of restriction policies from rank0 to rank3 in the generator. The settings for these policies are shown in Supplementary Table S3. We apply rank0 on day1 to 6, while apply rank1, rank2 and rank3 on day7, day8, and day9, respectively.

**Figure 5:**
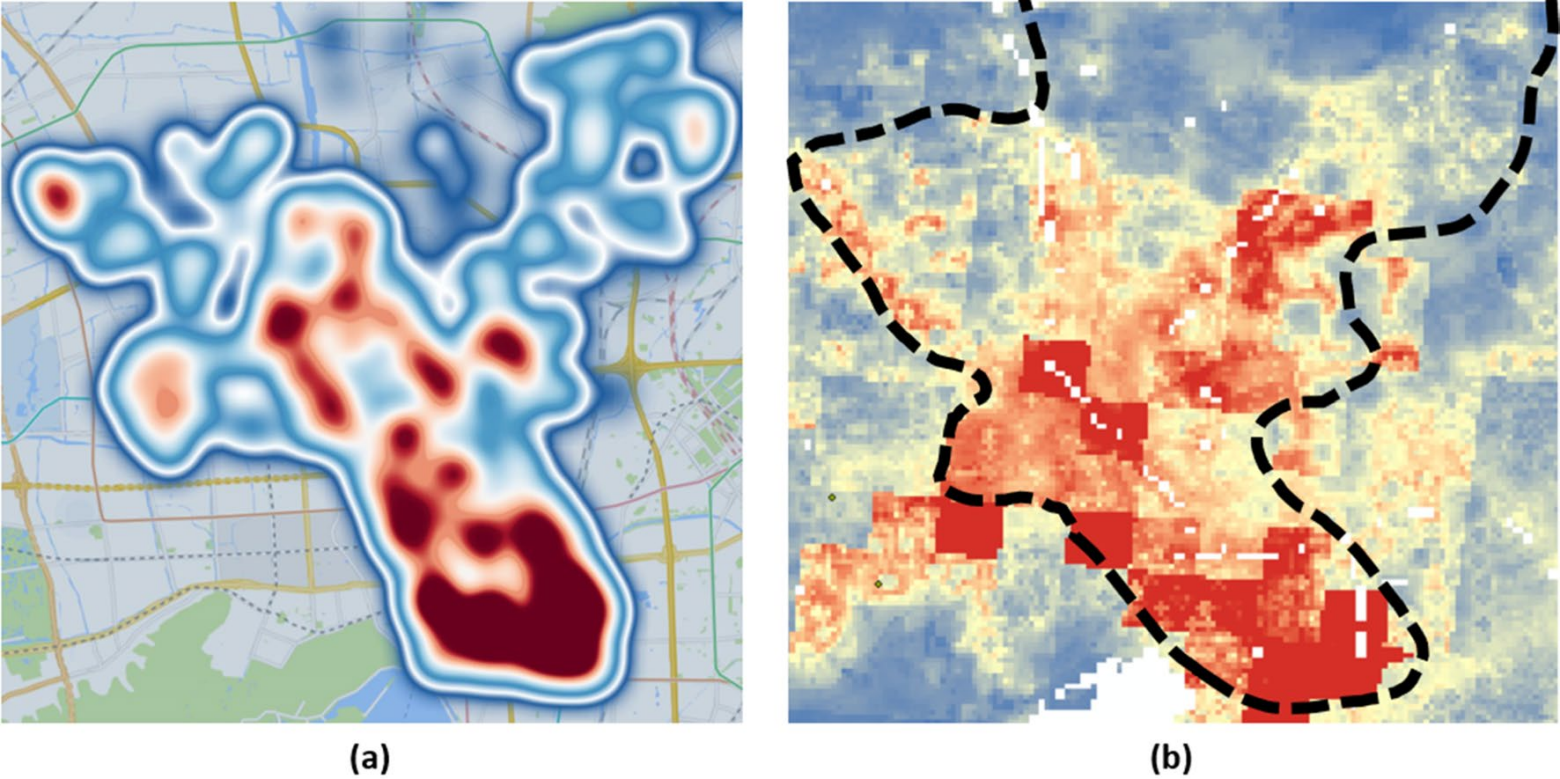
Comparing the starting point of our results with high-precision population density dataset published by Worldpop. (**a**) is the heat map based on the starting point of the trajectory we generated (created by Gaode Map JS API, version 2.0, https://lbs.amap.com/api/jsapi-v2/summary). (**b**) is the heat map based on the population density dataset published by Worldpop. We have marked the area we generated with black dashed lines e.

We used the generator to generate 10,000 agents. The trajectories for each agent were generated with a time range of 9 days in an urban district of Hangzhou. The start time of the trajectory dataset was 00:00 on March 1, 2022, and the end time was 23:59 on March 9, 2022. The policy settings were as in Supplementary Fig. S2. As a result, 3942 ordinary worker trajectories, 1045 overtime worker trajectories, 2509 retiree trajectories, 910 middle school student trajectories, 575 pupil trajectories, and 1019 freelancers trajectories were generated. In all the generated agents, the male to female ratio was 100.00:99.40, and the average age was 42.36. The age distribution of all the agents was shown in Supplementary Fig. S3. A heat map based on the location of the starting point of the generated trajectory is given in Fig. 5a. The generated results were compared with the high accuracy population distribution published by WorldPop^42^ (https://hub.worldpop.org/geodata/summary?id=49730) as shown in Fig. 5b, where the boundary of Gongshu District was indicated with the black dash line. As seen, the generated population shows the same pattern as the published data, which was dense in the southeast and sparse in the north.

**Figure 6:**
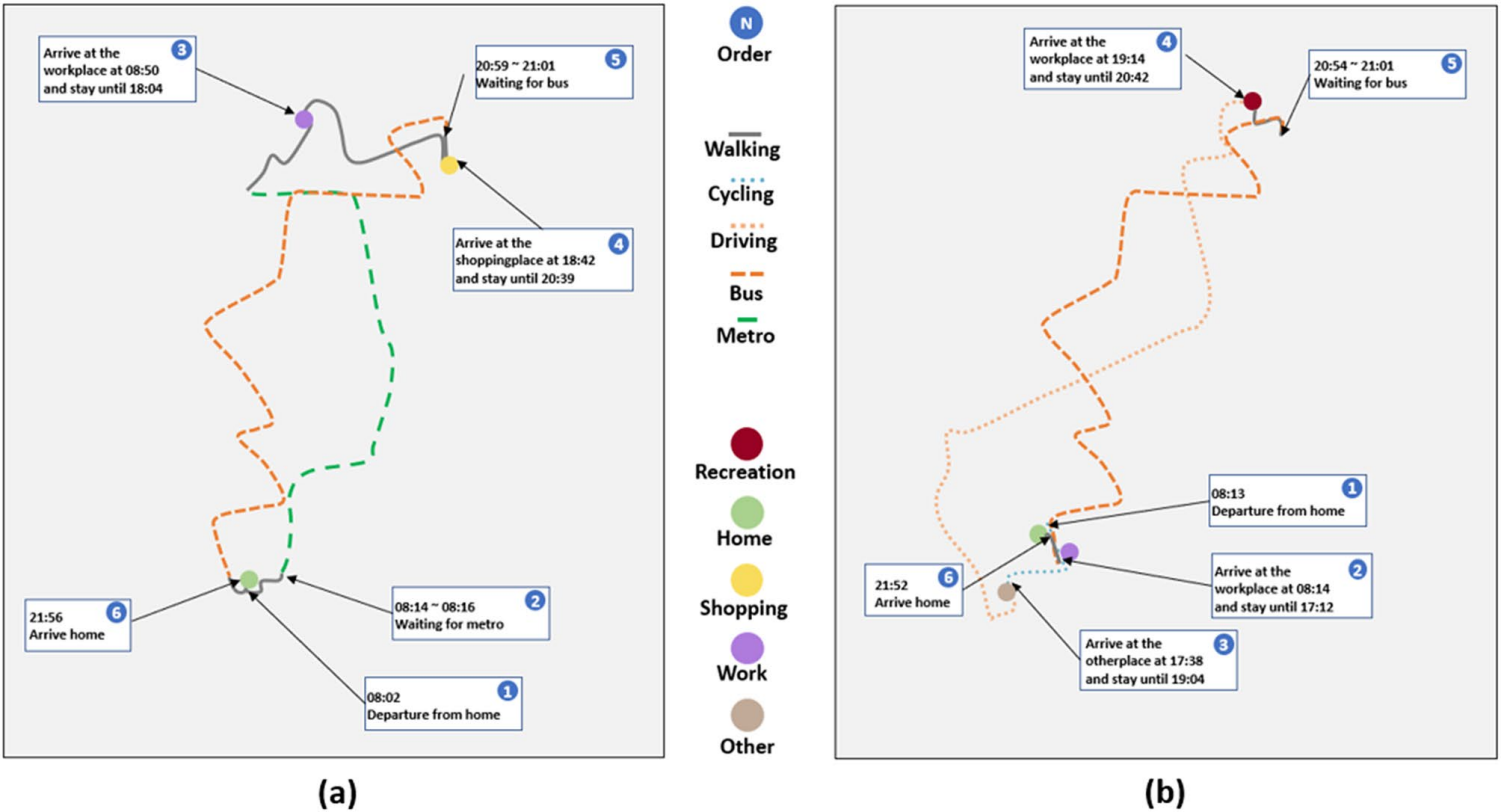
The trajectories of two agents (**a**, **b**) of ordinary worker group pattern on day1. (**a**, **b**) are the trajectories of two agents belonging to the ordinary worker we selected day1. The main reason for choosing these two agents is that they both use public transportation. We labeled the means of transportation they were passing and the time they stayed on the premises.

Figure 6 shows the generated trajectories of two ordinary workers on day1. Even though the two agents shared the same group pattern (with the same schedule template), the generated trajectories were different because each agent-specific schedule was unique. We noted that the workplace in Fig. 6a was farther from home than in Fig. 6b, so the agent in Fig. 6a used public transportation to travel to the workplace and spent 48 min on the trip. In contrast, Fig. 6b chose to ride to the workplace, which took only 1 min. We also noted that even though they arrived the bus station at different time (20:59 vs. 20:54, respectively), the agents in Fig. 6a and b depart by bus at the same time, which indicates that our method was able to simulate the shifts in public transportation while identify the agents travel in the same bus.

## Discussion

The radius of gyration can be used to measure the spatial distribution of human movement^43^. In general, a larger radius of gyration for an individual represents a wider range of activity. For each agent generated, the radius of gyration ${r}_{g}$ during the period $g$ is computed as follows:

4 $$\begin{array}{c}{r}_{g}=\sqrt{\frac{1}{n}\sum_{i=1}^{n}{\left|{\overline{a} }_{i}-{\overline{a} }_{c}\right|}^{2}}\end{array}$$

where $n$ is the number of places that stay in period $g$. ${\overline{a} }_{c}$ is the location of the center of mass calculated from all places. $\left|{\overline{a} }_{i}-{\overline{a} }_{c}\right|$ is the distance from the center of mass to each passing site.

**Figure 7:**
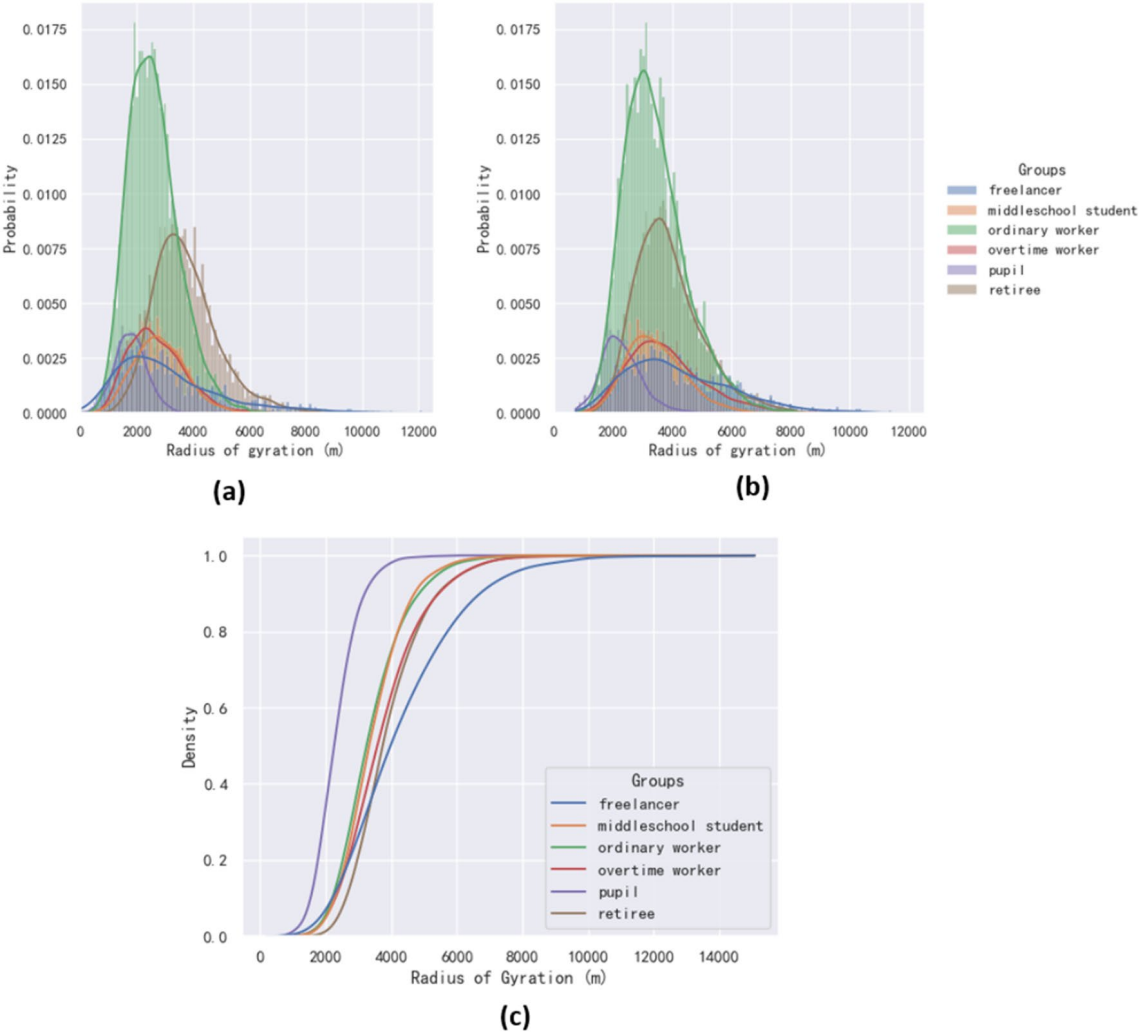
The radius of gyration of diverse groups on working days (**a**) and non-working days (**b**), and cumulative distribution function (CDF) of the radius of gyration for each groups (**c**). (**a**) The data from day1 to day4 were used to calculate the radius of gyration for working days. (**b**) The data from day5 and day6 were selected to calculate the radius of gyration for non-working days. (**c**) The data from day1 and day9 were selected to calculate the Cumulative distribution function (CDF) of the radius of gyration.

As the results, the curve in Fig. 7b was slightly right-shifted compared to Fig. 7a, which indicates that the activity range on non-working days is more abundant. This may because people go to farther places during holidays rather than the usually nearby workplaces. Notice that most of the group' radius of gyration is concentrated around 3 ~ 4 km and mostly less than 8 km, which were small compared to other similar studies^44^. The possible reason for this may because that we limited the trip area to Gongshu District in Hangzhou only, which made all the activities outside of the district were impossible. We could find in Fig. 7c that the pupil group had the steepest curve, indicating that the travel patterns of this group were highly similar. Middle school student, retiree, ordinary worker, and overtime worker had smooth curves, indicating that the heterogeneity of movement patterns within these four groups was greater compared to pupil group. The curves for Freelancer were the flattest, which means that their activities were the most irregular. This was consistent with the actual attributes for freelancers as more significant heterogeneity of group patterns.

**Figure 8:**
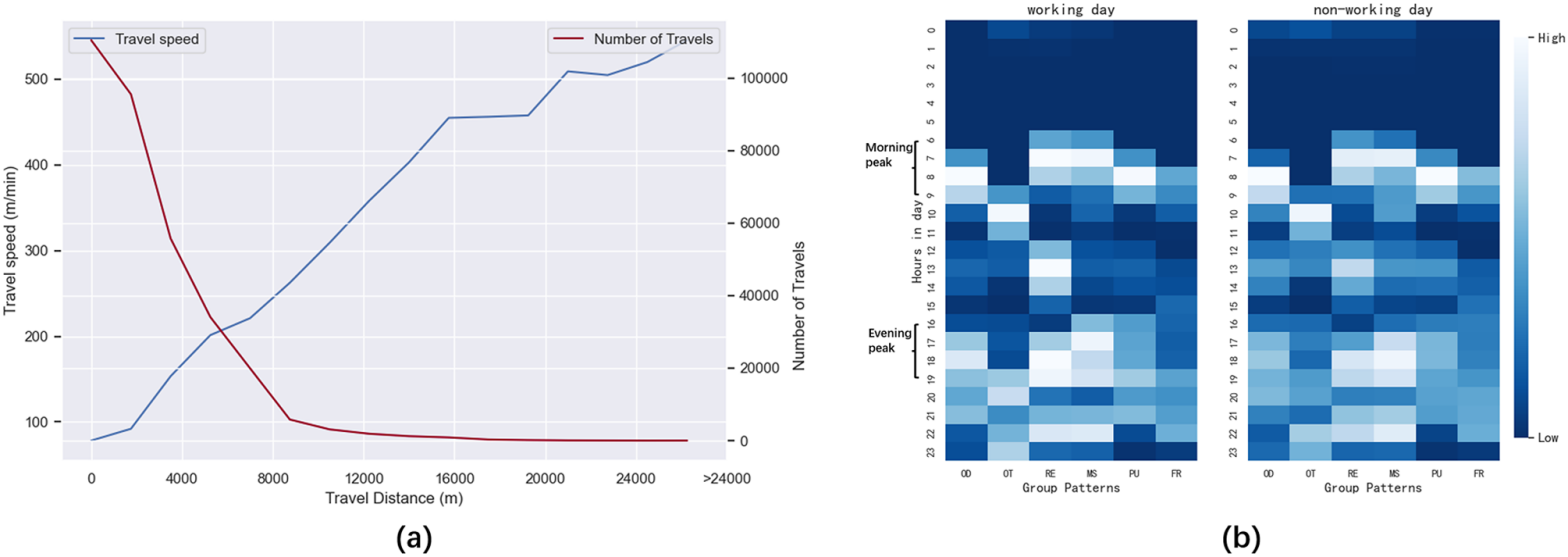
Trajectory statistics. Travel distance with travel speed and the number of travel (**a**) and Heat maps of the travel of different groups on working and non-working days (**b**). (**a**) We define all trips from a place to a new place as one trip, and since the places we set do not contain metro stations and bus stops, the duration of trips using public transportation is much higher. Distance refers to the straight-line distance between the origin and destination of a trip rather than the accurate distance traveled. (**b**) To facilitate comparison, we normalize the number of people in travel for each group and mark the morning peak and evening peak periods.

Figure 8a showed the relationship between travel distance with travel speed and the number of travels for all trajectories during day1 and day9. We noted that the number of travels is negatively correlated with the distance, which meant that most agent travels in a small-scale range, which was consistent with the real situation. The travel speed was positively proportional to the distance, which meant that our method enables the agent to choose the travel tool reasonably according to travel distance. We can estimate from Fig. 8a that most agents choose to walk for trips within 3 km, and as the distance increases, the agents choose to ride, drive, or by public transportation more often. In addition, the majority of the trips were within 10 km, and long trips over 25 km are rare. Figure 8b showed the heat map of different groups of people at different hours of the day. For most of the agents, the number of travel behaviors from 0 to 5 h is almost 0. It could also be found that the travel peaks of different populations differ.

**Figure 9:**
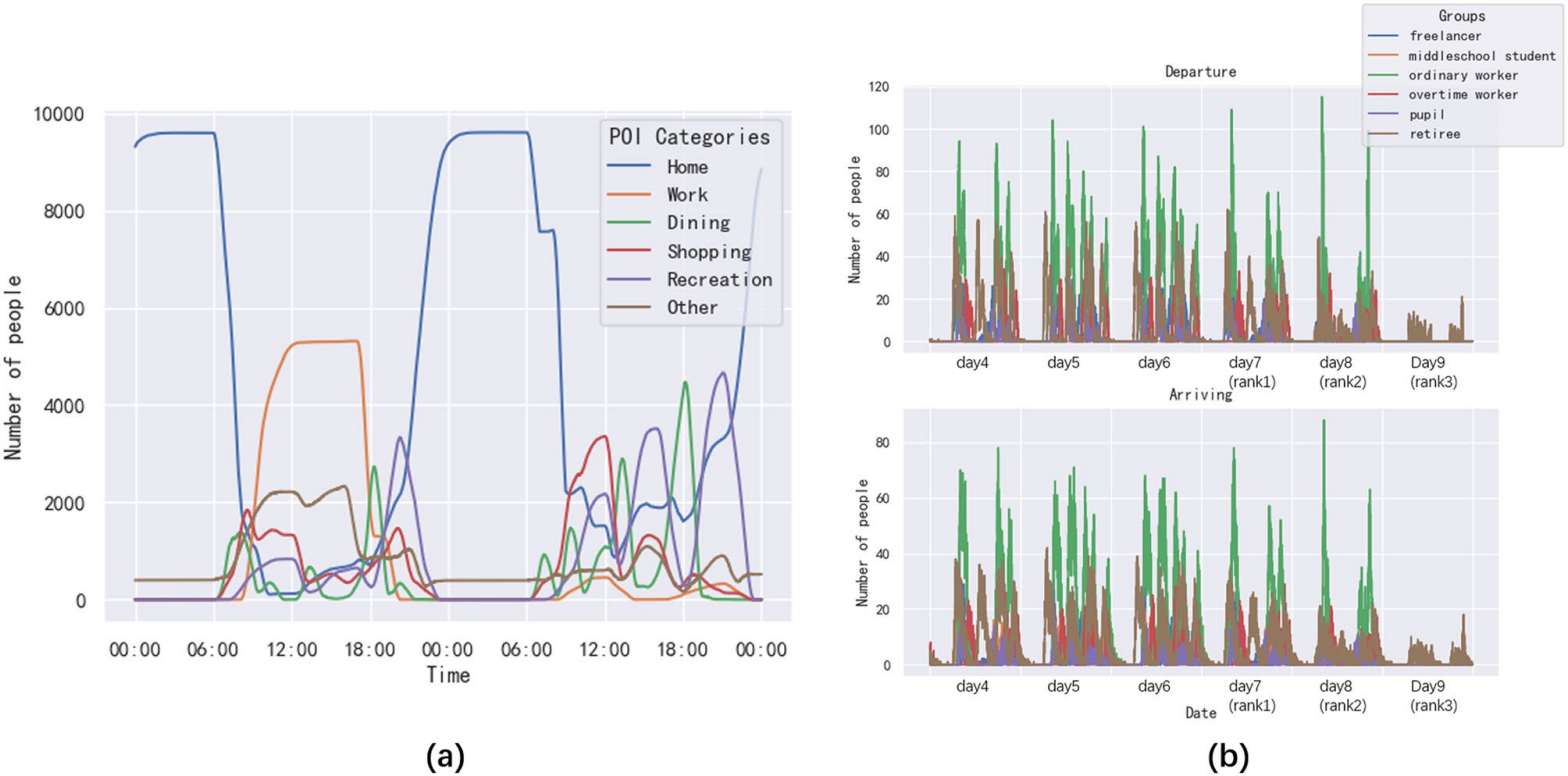
The number of people in different POI categories on day4 and day5 (**a**) and the impact of different restriction policies on the agent activities (**b**). (**a**) The main reason we chose day4 and day5 is that they are Friday and Saturday respectively, making it easy to compare. (**b**) We consider an agent leaving a place as a departure record and entering a place as an arriving record. We select day4 (working day), day5 and day6 (non-working days) as well as day7 (policy with rank1 implemented), day8 (policy with rank2 implemented), and day9 (policy with rank3 implemented) for the sake of comparison.

Figure 9a showed the number of people in different POI categories on day4 (working day) and day5 (non-working day). In the early morning of day4, almost all the agents were resting at home. After about 6:00 am, agents left home and the number of people at home dropped rapidly, while the number of people in other places gradually increased. Since day4 was a working day, most agents go to work, so the rise in the workplace was the most rapid. We noticed that the peak for dining places was in the morning, noon and evening, regardless of working days or non-working days. Compared to working days, the number of people at consumption places and entertainment places on non-working days rise sharply, while the number of people at workplaces decreased substantially. The above-observed information was consistent with our setting as well as people's common patterns.

Figure 9b showed the effect of different levels of the NPIs on travels from day4 to day9. We noted that agents' activities showed a pattern of two peaks regardless of working and non-working days, i.e., in the morning from about 7 am when many agents depart, and in the evening again around 5 pm. For day7, when rank1 was implemented, there was no significant change from the curve, and only a small number of trips were restricted. For day8, when rank2 was implemented, dining was no longer available, as were recreational, public, and educational activities, and the probability of traveling to work was reduced by 30%. We could note the decrease in student trips in the curve and the decrease in evening peak trips. It was worth noting that there was still a peak in the evening of the day8, which is unrealistic because our current policy simulation did not change the schedule itself. Most trajectories return home in the evening. On day9, when rank3 was applied, almost no one goes out, and only some retirees who were allowed to go to the hospital as set in the schedule. The impact of the policy intervention was especially obvious for pupils and middle school students because the schools were closed. As a result, our method could simulate the responses of different groups of people to the same policy.

In our current results, only 10,000 agents were simulated within Gongshu District, Hangzhou, which was relatively smaller amount comparing to the actual population in the area. To evaluate the scale-up potential of our method, several experiments were performed. For the scale-up of number of generated, we recorded the consuming time for different number of generated, see Supplementary Fig. S4. As a result, the time-consuming increases as the number of agents grew when sequential process was performed for each agent. However, high proportion of the total time consumption (around 97%) was on the response time from the LBS provider. If asynchronous requests are used, there will be an order of magnitude improvement in the generation speed. On the one hand, because LBS-based method provides access to geographical data in various levels, our simulation can be scaled up in geographic scope. We evaluated the consuming times for trajectory generation with maximum distances of 10 km, 100 km, and 1000 km to mimic the trips of city, provincial and country level. The average time consumptions were 0.27 s, 0.35 s and 1.02 s, respectively (see Supplementary Table S4). Such results indicate that the time consumption did not increase linearly as the distance increases. Therefore, when the geographic scope is scaled up, its effect on the generation time is acceptable. Moreover, even at larger scales than cities, the majority of trips are concentrated within 100 km. In summary, for both needs for an increasement on agent quantitative as well as geographic scope, our approach had the potential to scale-up to the full-scale. Given the problem of service coverage of LBS providers (a single LBS provider may only provide services for part of the countries), it may also be necessary to integrate data from multiple LBS providers if the population at the state-level is to be generated.

In this paper, we proposed a method for generating pseudo-human mobility datasets using location-based services (LBS), which had advantages over other studies in similar fields. For example, Ban^45^ et al. implemented an agent-based simulator for Covid-19, in which different activity schedules were assigned for three age categories (children, adult, and retired people).Therefore, there were only limited number of daily patterns of activity chains for agents, Liao^46^ et al. proposed a simulation method which also used predefined activity patterns for Covid-19 transmission simulation, and the activity location of the same type was selected randomly, which was similar to the strategy utilized in our work. However, their simulation was restrained to on-campus locations, and the daily activity pattern covers only weekdays. In Kumar^47^ et al., daily activity schedules were simulated for each individual including activities of home, work, education, shopping or other. Trip trajectories between each two consequent activities were generated according to road and transit networks, similar as ours. However, their simulation was at a 5-min resolution, while ours was in each 5-s. In conclusion, our method was able to generate pseudo population mobility data with high temporal and spatial resolution using only predefined population patterns and data from LBS, which was very helpful for studies related to infectious disease modeling. To the best of our knowledge, this is the first attempt to used LBS to generate pseudo population mobility data.

## Conclusion

In this paper, we proposed a method to generate pseudo mobility data on a per-agent basis. To validate our method, we generated 10,000 agents’ travel records with a starting point in a district of Hangzhou. The mobility data was analyzed with the distribution of the starting point, the radius of gyration, the travel situation, and the number of people in the premises, and it was concluded that the generated data is in accord with the urban-scale group patterns. Our method could generate not only agent-based mobility data, but also Covid-19-related attributes of the agents, therefore could be used for ABM-based epidemic simulation. In addition, We have implemented a web-based trajectory generator. This allowed the user to configure various parameters to meet the needs of different researches without any code-level changes. However, there were limitations in our current work. First, the agents generated were lightweight in numbers and are restricted at city district level. However, we discuss the potential of our method to scale up in both geographic and agent quantity aspects. Second, the focus of our current work was on the feasibility of generating agent-based daily mobility data by using LBS to acquire geographic and road map information. Although our method included a component of synthetic population generation, it was based on rough calculation. In the future, techniques including Synthetic Reconstruction (SR) or the Combinatorial Optimization (CO) will be adapted. Besides, instead of random selection from POIs returned by the LBS, how to select the agent-specific POI in a more reasonable way will be explored in our future work.

## Supplementary Information

Supplementary Information.

## Data Availability

The population distribution dataset that we used to compare with our results is publicly available from WorldPop at https://hub.worldpop.org/geodata/summary?id=49730. All other data included in this study are available upon request by contact with the corresponding author. The dataset we generated is available athttps://drive.google.com/drive/folders/1a4KCyat7MdP6CeJvcV1CwvTmYL1g-woC?usp=sharing.
