# Supplementary material for: An LBS and agent-based simulator for Covid-19 research
Source: Sci Rep. 2022 Dec 8;12:21254. doi: 10.1038/s41598-022-25175-5 (PMC9731980; doi:10.1038/s41598-022-25175-5)
Supplement: Supplementary file 1 — Supplementary Information. [file 41598_2022_25175_MOESM1_ESM.docx]

### Supplementary

**Supplementary Figure S1** The user interface for the data generator


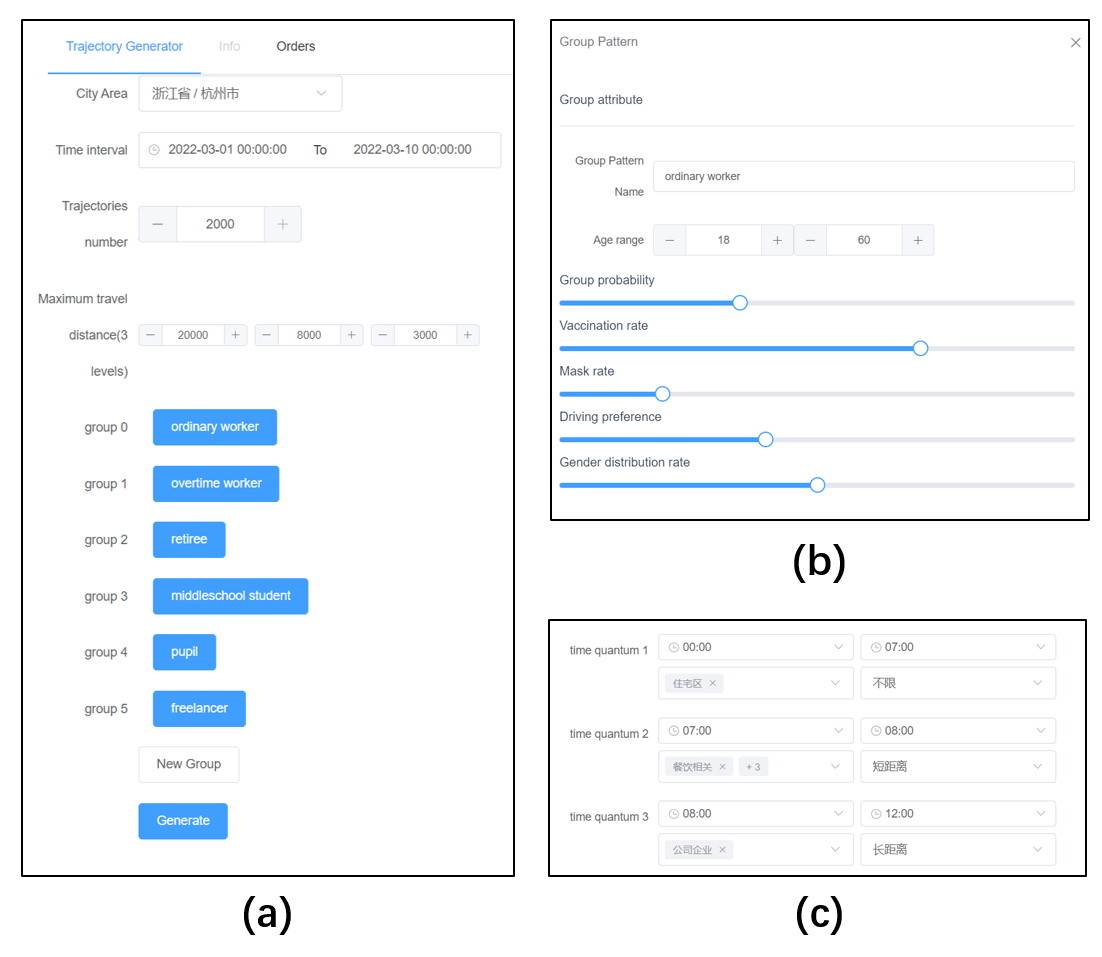


**Supplementary Figure S2** The date range of the generated trajectories and the corresponding restriction policies.


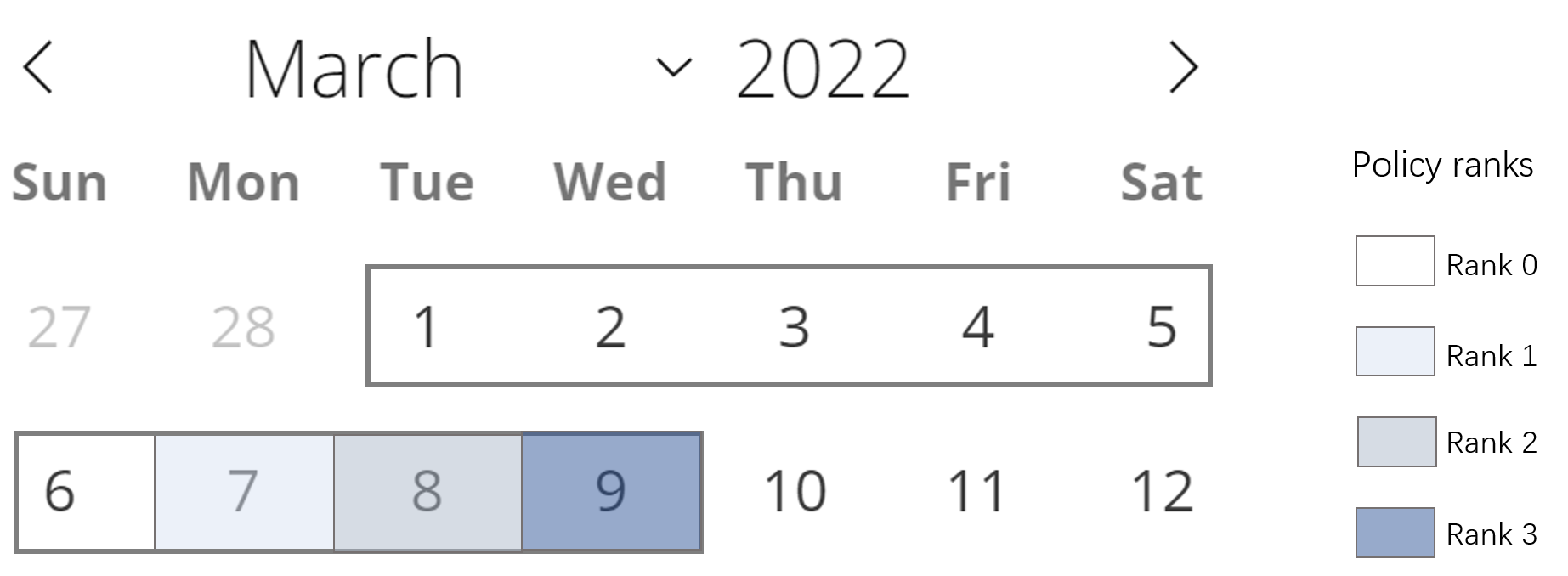


**Supplementary Figure S3** Age distribution of generated agents。


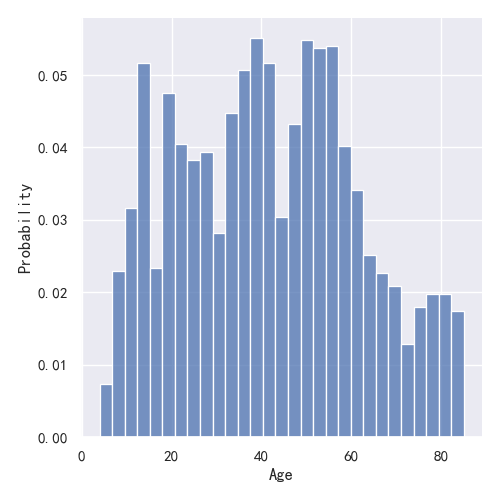


**Supplementary Figure S4** Relationship between the number of generators and the total time spent and the percentage of waiting time.


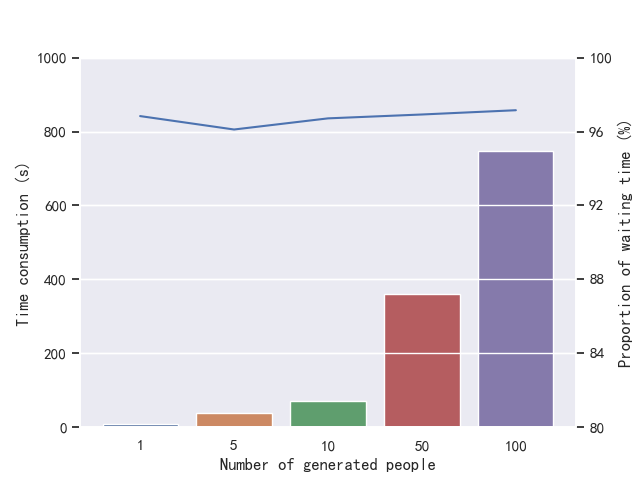


**Supplementary Table S1.** GPS trajectory data recorded at each time.

| Name | format | Description |
| --- | --- | --- |
| Time stamp | Integer e.g. 2022-03-09 23:56:58 | The time of the location |
| Longitude | Float e.g. 120.177014 | The longitude of a certain moment |
| Latitude | Float e.g. 30.302263 | The latitude of a certain moment |
| Typecode | String e.g. 120300 | Type code for POI can be used in the Gaode Open Platform. Ways of travelling if not within the POI. E.g. walking |
| POI category | String e.g. home | POI Categories we define |
| POI ID | Stirng e.g. B023B08Y4C | The Code in the Gaode Open Platform can correspond to a unique POI. -1 if not within the POI |
| Mask status | Integer e,g, 1 | 1 means wearing a mask and 0 means not wearing a mask |

**Supplementary Table S2.** Settings of group patterns.

| Group | Group rate (%) | Vaccination rate (%) | Mask rate (%) | Age range | Gender distribution | Driving preference | have non-working day |
| --- | --- | --- | --- | --- | --- | --- | --- |
| Ordinary worker | 40% | 70% | 20% | 18~60 | 5(1:1) | 4 | yes |
| Overtime worker | 10% | 70% | 30% | 18~60 | 5(1:1) | 5 | yes |
| retiree | 25% | 70% | 20% | 50~85 | 5(1:1) | 2 | no |
| Middle school student | 9% | 80% | 50% | 12~18 | 5(1:1) | 1 | yes |
| pupil | 6% | 90% | 40% | 6~13 | 5(1:1) | 0 | yes |
| freelancer | 10% | 60% | 30% | 18~60 | 5(1:1) | 6 | no |

**Supplementary Table S3.** Settings of different restriction policies

| Rank of restrictions | policy description |
| --- | --- |
| Rank0 | No restrictions. |
| Rank1 | Nighttime recreation is prohibited. Halve the capacity of dining and public shopping. |
| Rank2 | Nighttime recreation, daytime recreation, public shopping, public science and education, dining are prohibited. The capacity of work is reduced by 30% and the capacity of normal shopping is halved. The mask rate of all agents was increased to 80% |
| Rank3 | Except medical place and home. All type were prohibited. |

### **Supplementary Table S4.** Relationship between distance traveled and average time spent

| Traveling distance (km) | Average time consumption (s) |
| --- | --- |
| 10 | 0.27 |
| 100 | 0.35 |
| 1000 | 1.02 |
